# The Unsaturated/Saturated Fatty Acid Ratio: A Metabolic Hub and Therapeutic Vulnerability in Glioblastoma

**DOI:** 10.3390/biomedicines14081757

**Published:** 2026-08-04

**Authors:** Xuhao Dai, Jialin Ku, Haixiang Li, Runxi Yan, Baofeng Wang

**Affiliations:** 1 Department of Neurosurgery, Tongji Hospital, Tongji Medical College, Huazhong University of Science and Technology, Wuhan 430030, China; xuhao_dai@163.com (X.D.); m202376550@hust.edu.cn (J.K.); d202582828@hust.edu.cn (H.L.); yan18339227567@163.com (R.Y.); 2 Junshan Brain Center, Tongji Hospital, Tongji Medical College, Huazhong University of Science and Technology, Wuhan 430030, China

**Keywords:** glioblastoma, fatty acid metabolism, unsaturated/saturated fatty acid ratio

## Abstract

Glioblastoma (GBM) exhibits profound metabolic reprogramming, among which the balance between unsaturated and saturated fatty acids (UFA/SFA) emerges as a critical determinant of tumor behavior and treatment response. Recent studies have shown that fatty acid metabolic reprogramming is a key mechanism driving GBM progression. Abnormalities in fatty acid uptake, synthesis, desaturation, and oxidation collectively reshape the lipid composition of tumor cells, particularly by altering the unsaturated/saturated fatty acid ratio. Monounsaturated fatty acids mainly promote tumor cell proliferation and membrane biosynthesis, polyunsaturated fatty acids can induce lipid peroxidation and ferroptosis under specific stress conditions, whereas excessive saturated fatty acids can cause lipotoxicity when desaturation is limited. Key enzymes in fatty acid metabolism constitute a regulatory network and provide potential therapeutic targets for GBM. In addition, fatty acid metabolism can remodel the tumor immune microenvironment, especially by affecting the functional state of tumor-associated macrophages. Rather than targeting a single lipid species or isolated metabolic enzyme, therapeutic strategies that recalibrate the UFA/SFA ratio may provide a more integrated approach to restraining GBM progression and improving treatment sensitivity.

## 1. Introduction

Glioblastoma (GBM) is one of the most common and biologically aggressive tumors of the adult nervous system, with rapid growth, strong invasiveness, and marked treatment resistance. Even with maximal safe resection combined with chemoradiotherapy and tumor treating fields, the median survival of patients remains less than two years [1,2]. With continued research, it has become increasingly recognized that, in addition to classic glycolytic reprogramming, GBM also exhibits marked lipid metabolic abnormalities, including multiple processes such as fatty acid uptake, de novo synthesis, chain elongation, desaturation, storage, and oxidative utilization; together, these processes participate in the core network governing tumor growth, membrane synthesis, redox homeostasis, and therapy resistance [3]. Chemically, fatty acids can be classified by the number of double bonds into saturated fatty acids (SFA; no double bond), monounsaturated fatty acids (MUFA; one double bond), and polyunsaturated fatty acids (PUFA; two or more double bonds) [4]. These structural differences not only determine the arrangement of fatty acids in cellular membranes, but also affect membrane fluidity, susceptibility to lipid peroxidation, and the activity of related signaling pathways [5,6]. Multi-omics studies have shown that different molecular subtypes of GBM display distinct lipidomic profiles, and the lipid composition of tumor tissue differs markedly from that of normal brain tissue, particularly in PUFA-related phospholipids, very-long-chain fatty acids, and glycerolipids [7]. This suggests that fatty acid compositional imbalance is not merely an accompanying phenomenon, but may directly influence the biological behavior of glioma, differences in molecular subtyping, and divergence in treatment response. Therefore, this review focuses on the sources, metabolism, and functions of MUFAs, PUFAs, and SFAs in glioblastoma, with a central theme that the imbalanced UFA/SFA ratio acts not merely as a metabolic consequence but as a master regulator integrating membrane biology, signal transduction, cell fate decisions, and immune modulation in GBM. It further emphasizes how this ratio affects glioma proliferation, invasion, ferroptosis sensitivity, remodeling of the immune microenvironment, and clinical treatment response, and discusses potential translational directions to provide a theoretical basis for GBM treatment.

## 2. Effects of Unsaturated and Saturated Fatty Acids on GBM Phenotypes

### 2.1. Cell Proliferation and Metabolic Reprogramming

GBM exhibits pronounced lipid metabolic reprogramming, one of its core features being a dual dependence on fatty acids as both energy substrates and structural molecules. Tumor cells maintain sustained proliferation in hypoxic and nutrient-restricted environments through fatty acid uptake, storage, and beta-oxidation [8]. In GBM, the formation of abundant lipid droplets is regarded as an important hallmark of fatty acid metabolic reprogramming. These lipid droplets can release fatty acids through autophagy, thereby maintaining energy homeostasis and promoting tumor cell survival and growth [9]. Inhibition of key enzymes involved in fatty acid oxidation can significantly suppress GBM growth, indicating that fatty acids are not only membrane components, but also important energy sources driving tumor proliferation [10].

Among different fatty acids, MUFAs are one of the most direct lipid substrates supporting GBM proliferation, with oleic acid (C18:1) being the most representative example [11]. Taïb B et al. found that adding oleic acid to U138 GBM cells induced the accumulation of lipid droplets containing oleic-acid-enriched triglycerides, while simultaneously enhancing fatty acid oxidation and glucose metabolism, ultimately markedly increasing glioma cell proliferation [12]. Although established GBM cell lines such as U138 are widely used for mechanistic studies of lipid metabolic regulation, their metabolic characteristics may not fully capture the heterogeneity of patient tumors, and findings from these models should be interpreted together with evidence from patient-derived systems. Another study showed that oleic acid can bind fatty acid-binding protein 7 (FABP7), promote its nuclear translocation, and induce nuclear lipid droplet formation, thereby upregulating the transcription of proliferation-related genes through regulation of histone acetylation and ultimately driving glioma cell proliferation [11]. FABP7 is highly expressed in glioma stem cells and participates in the intracellular transport of PUFAs and MUFAs; this “fatty acid chaperone” function enhances the support of fatty acids for glioma stemness and proliferative capacity [13]. At the metabolic level, MUFA generation depends on desaturases such as SCD1/5. This process not only provides substrates for membrane lipid synthesis, but also prevents lipotoxicity caused by SFA accumulation, and is therefore considered a key step in maintaining lipid homeostasis in tumor cells [14].

The effects of PUFAs on glioma cells are more complex. In GBM, their role shows a “double-edged sword” effect, as they can participate in membrane construction while also inducing cellular injury under specific conditions [8]. On the one hand, PUFAs can be incorporated into phospholipids and regulate membrane fluidity and receptor function, thereby promoting signal transduction [15]; on the other hand, exogenous PUFAs, especially omega-3 fatty acids, can disrupt lipid homeostasis, induce lipid droplet breakdown and the accumulation of free fatty acids, and subsequently trigger lipid peroxidation and inhibit tumor growth [8]. For example, the functions of omega-6 PUFAs (such as arachidonic acid, AA) and omega-3 PUFAs (such as docosahexaenoic acid, DHA) in tumor cells are opposite [16]. Similar evidence has also been reported in other tumors, where omega-3 PUFAs have been shown to inhibit PI3K/Akt signaling and suppress tumor growth in breast cancer and colorectal cancer [17,18].

Unlike unsaturated fatty acids, SFA in GBM more often represents a form of “metabolic stress,” and whether it can be converted to MUFA in time determines its effect [19]. When the activity of desaturases such as SCD1 is limited, intracellular accumulation of SFA (such as palmitic acid, PA) can induce endoplasmic reticulum stress, mitochondrial dysfunction, and apoptosis, a phenomenon known as lipotoxicity [20]. In GBM, the lipid metabolic system rapidly converts SFA into MUFA or stores it in lipid droplets to avoid this toxicity, thereby maintaining the proliferative capacity of the cells [21]. Reports have indicated that brain tumor tissues are rich in unsaturated fatty acids but relatively deficient in SFA, suggesting that tumor cells are more inclined to accumulate unsaturated fatty acids [12]. Therefore, from the perspective of metabolic balance, SFA itself is not the main driver of proliferation; rather, it indirectly supports tumor growth by being converted into MUFA, which explains why changes in the SFA/MUFA ratio are highly important for tumor fate.

Overall, the roles of MUFAs, PUFAs, and SFAs in GBM are not isolated; instead, they form a dynamic balance system: MUFAs mainly promote proliferation and membrane synthesis, PUFAs can induce metabolic collapse and cell death under specific conditions, whereas SFAs are continuously regulated as metabolic intermediates between toxicity and utilization. Changes in fatty acid composition and in the UFA/SFA ratio not only determine cellular energy supply but also affect epigenetic regulation, membrane architecture, and signaling networks, thereby jointly driving metabolic reprogramming and malignant progression in glioma. Notably, the molecular structure of fatty acids (chain length and double-bond position) also affects their arrangement in the cell membrane, thereby modulating membrane fluidity and receptor function [15,22,23]. Critically, the net effect on GBM cells is not determined by any single fatty acid species, but by the UFA/SFA ratio. A high MUFA/SFA ratio favors proliferation, survival, and membrane biosynthesis, whereas a high PUFA/SFA ratio predisposes cells to lipid peroxidation and ferroptosis under oxidative or therapeutic stress.

### 2.2. Cell Apoptosis and Therapy Resistance

Glioma cells are highly sensitive to changes in fatty acid composition, and lipid peroxidation is a key hub linking fatty acid metabolism to cell death. Its dysregulation can simultaneously drive apoptosis, ferroptosis, and changes in treatment response [24]. The main source of lipid peroxidation is PUFA-enriched membrane phospholipids. Because these lipids contain multiple double bonds, they are highly susceptible to free radical chain reactions under oxidative stress, thereby disrupting membrane integrity and inducing cell death [25]. Lipid peroxidation is a driving force of ferroptosis, and PUFAs are incorporated into phospholipids through the action of related enzymes, forming oxidizable PUFA-PE, which is the critical substrate basis for ferroptosis [17,26,27]. In this process, lipid peroxidation not only directly damages membrane structure, but also exacerbates reactive oxygen species (ROS) accumulation and further activates the mitochondrial apoptosis pathway, forming a positive feedback loop of “peroxidation-death” [6,24].

PUFAs in GBM exhibit a typical “pro-death effect,” especially under exogenous supplementation or treatment-induced conditions [27]. Studies have shown that increasing intracellular PUFA levels can markedly enhance lipid peroxidation, thereby inducing ferroptosis and inhibiting tumor growth [28]. At the same time, in the context of chemoradiotherapy, PUFA-driven lipid peroxidation can amplify ROS-mediated damage and thus enhance tumor cell killing, suggesting potential value for treatment sensitization [29]. For example, Kant et al. found that under radiotherapy, a PUFA-rich diet (especially omega-3) caused lipid droplets in tumor cells to rupture and release free fatty acids, increasing lipid peroxidation and thereby enhancing the cytotoxic effect of radiotherapy on glioma [8]. However, glioma cells possess escape mechanisms, such as downregulating acyl-CoA synthetase long-chain family member 4 (ACSL4) to reduce PUFA incorporation or upregulating antioxidant systems such as glutathione peroxidase 4 (GPX4) to eliminate lipid peroxidation products, thereby suppressing PUFA-mediated cell death [27,30]; this process is also considered closely related to temozolomide (TMZ) resistance [31]. Therefore, the function of PUFAs depends on the dynamic balance between “peroxidation burden” and “antioxidant capacity.”

Unlike PUFAs, MUFAs mainly act as “protective lipids” in the regulation of cell death, reducing lipid peroxidation by competitively replacing PUFAs in membrane phospholipids [25]. Studies have shown that MUFAs (such as oleic acid) can significantly reduce the proportion of PUFAs in the membrane, thereby decreasing the sites attacked by ROS and inhibiting ferroptosis [32]. In GBM, this reduction in membrane PUFA content often depends on the activity of desaturases such as SCD1, which convert SFA into MUFA, thus maintaining membrane fluidity while avoiding excessive oxidative damage [20]. Therefore, MUFAs not only participate in proliferation, but also play a key role in suppressing lipid peroxidation and maintaining cell survival.

By contrast, SFA in GBM can either induce cell death or, under specific conditions, contribute to the development of resistance [20]. On one hand, when SFA (such as palmitic acid) accumulates excessively and cannot be desaturated, it can induce endoplasmic reticulum stress, mitochondrial injury, and ROS generation, thereby triggering apoptosis or non-apoptotic cell death (lipotoxicity) [33]. On the other hand, in the context of metabolic reprogramming, SFA can be rapidly converted into MUFA or stored in lipid droplets, thereby avoiding toxic accumulation and indirectly enhancing cell survival [34,35]. Parik S et al. reported that inhibiting desaturases so that palmitic acid cannot be converted into unsaturated fatty acids leads to intracellular accumulation of palmitic acid and induces apoptosis; this accumulation of SFA has a synergistic effect with radiotherapy and TMZ chemotherapy and can re-sensitize TMZ-resistant glioma cells [31]. More importantly, SFA is also involved in protein palmitoylation, a modification that regulates the localization and signaling activity of key receptors such as EGFR, thereby affecting cellular responses to radiotherapy and chemotherapy [34,35].

Overall, fatty acids determine the sensitivity of glioma cells to treatment by regulating the axis of “lipid peroxidation-antioxidant defense-cell death.” When PUFAs predominate, cells are more prone to lipid peroxidation and to entering ferroptotic or apoptotic programs, thereby increasing treatment sensitivity; when the MUFA ratio rises or antioxidant systems are activated, cells can more effectively remove lipid peroxidation products and thus acquire resistance to chemotherapy and radiotherapy [26]. The impact of fatty acid ratios on death sensitivity has been validated in multiple tumors. For example, in hepatocellular carcinoma and breast cancer, upregulation of SCD1 increases the MUFA ratio and significantly suppresses ferroptosis while promoting drug resistance [36,37]. In summary, MUFAs, PUFAs, and SFAs do not independently regulate cell fate; rather, they jointly determine the death threshold and treatment response of glioma cells through dynamic balance, providing an important theoretical basis for targeting fatty acid metabolism to overcome resistance. Thus, the biologic consequence of fatty acid remodeling is best interpreted as a balance problem: MUFA enrichment raises the threshold for lipid-peroxidation-mediated death, whereas PUFA enrichment lowers this threshold and may sensitize GBM cells to radiotherapy, chemotherapy, or ferroptosis-inducing interventions.

### 2.3. Cell Migration/Invasion and Stemness

The migration and invasion of glioma cells are not simply due to increased cellular motility, but result from the combined effects of fatty acid uptake, transport, oxidative energy production, and membrane lipid remodeling. Fatty acid metabolism promotes stronger infiltrative capacity and adaptability of glioma cells by providing energy and structural support for cytoskeletal rearrangement, adhesion transitions, and distal survival [38].

Among unsaturated fatty acids, FABP7 is a key hub regulating invasion. Nuclear localization of FABP7 is more common in infiltrative glioma and is closely associated with poor prognosis in EGFR-overexpressing GBM. Inhibition of FABP7 in neurosphere-like glioma cells can simultaneously weaken proliferation and migration, indicating that this fatty acid-binding protein is not only involved in fatty acid transport, but also in maintaining invasive phenotypes [39]. DHA and AA show opposite effects on migration in glioma: DHA binds FABP7 and retains it in the nucleus, thereby inhibiting migration, whereas AA binding to FABP7 promotes migration by upregulating COX-2 and downregulating PPARγ. This indicates that the determinant of phenotype is not the absolute amount of a single fatty acid, but rather the internal balance of PUFAs and their coupling with FABP7 [16]. Glioma stem cells are also highly dependent on FABP7; their highly expressed neurosphere-like cells more closely resemble the invasive phenotype of the primary tumor [40], and FABP7-related fatty acid transport capacity helps maintain the adaptability and heterogeneity of the stem cell pool [41]. In other tumors, DHA similarly suppresses invasion and migration, whereas AA can promote motility through lipid raft-associated receptors and lipoxygenase pathways [42].

Compared with unsaturated fatty acids, direct evidence for MUFAs in glioma migration is relatively limited. However, studies in other tumors have shown that oleic acid can promote migration and invasion of breast cancer through the FFAR1/4-EGFR-AKT axis, and that CD36 preferentially takes up MUFAs during cell detachment to buffer palmitic acid toxicity and maintain metastatic potential [43,44]. Therefore, in GBM, MUFAs are more likely to indirectly enhance invasiveness by maintaining membrane fluidity, buffering SFA toxicity, and supporting adhesion/cytoskeletal signaling. In addition, there is evidence that MUFAs help maintain stemness; Hale JS et al. reported that in high-fat diet models, especially those rich in MUFAs, tumor stem cells increased significantly, tumor growth accelerated, and adaptability to ischemia and necrosis became stronger [45].

Unlike unsaturated fatty acids, SFA (such as palmitic acid) can reduce cardiolipin in glioma cell mitochondria and promote cytochrome c release, thereby activating the mitochondrial apoptosis pathway [46]. In other tumors, palmitic acid may either inhibit growth and metastasis or avoid lipotoxicity through CD36-mediated MUFA compensation [46]. At present, research on saturated fatty acids in GBM has focused mainly on their lipotoxic effects when present in excess. SCD1 maintains endoplasmic reticulum homeostasis and tumor-initiating capacity in glioma stem cells, and inhibition of SCD1 leads to SFA accumulation, induces ER stress and apoptosis, and impairs RAD51-mediated DNA repair [20]. Recent studies have also shown that SCD1 and the brain-specific isoform SCD5 have non-redundant desaturase functions in GBM, and together support stem cell maintenance and genomic stability. This indicates that glioma cells rely on desaturation programs to reduce SFA in order to sustain invasive survival advantages [20]. In addition to synthesis and desaturation, fatty acid oxidation (FAO) is also involved in GBM infiltration. Acyl-CoA binding protein (ACBP) maintains GBM invasion by enhancing FAO; meanwhile, FAO can also drive radioresistance and CD47-mediated immune evasion, suggesting that invasiveness, stemness maintenance, and therapy resistance share the same lipid metabolic reprogramming network [10,47].

## 3. Expression and Mechanisms of Key Fatty Acid Metabolic Enzymes in GBM

The phenotypic consequences of fatty acid imbalance described above are closely linked to the activity of key metabolic enzymes that regulate fatty acid synthesis, desaturation, elongation, and oxidation. Fatty acid metabolic reprogramming in GBM is essentially a continuous reaction network centered on “acetyl-CoA supply-de novo fatty acid synthesis-fatty acid desaturation-long-chain fatty acid elongation,” and each key enzyme determines whether tumor cells can maintain membrane lipid renewal and growth advantages under hypoxia, nutritional fluctuations, and therapeutic stress [48]. Glioma cells meet the demand for fatty acids during rapid proliferation by upregulating fatty acid synthesis and desaturation enzymes, including ATP citrate lyase (ACLY), acetyl-CoA carboxylase (ACC), fatty acid synthase (FASN), desaturases (such as SCD1, SCD5, FADS1/2), and fatty acid elongases (the ELOVL family) [22,49,50].

At the upstream end of this network, ACLY converts citrate into cytosolic acetyl-CoA. In GBM cells, ACLY is enriched not only in pseudopod-like structures, but also in the increase in acetyl-CoA, which can promote cell adhesion and migration through H3K27ac remodeling and Ca^2+^-NFAT1 signaling [51]. Further downstream, EGFRvIII, PI3K/Akt, and mTORC1 can translate oncogenic signals into a lipogenic transcriptional program through SREBP-1, driving expression of ACLY, ACC, FASN, and SCD, thereby stably converting proliferative signals into fatty acid synthesis flux [52]. In GBM, the SOAT1-SREBP-1 axis can regulate the lipogenic program through a feedback mechanism involving lipid droplets and cholesteryl esters, indicating a clear transcriptional basis for GBM dependence on fatty acid synthesis [53]. ACC1/ACC2 are key gatekeepers that convert acetyl-CoA into malonyl-CoA. In EGFRvIII glioma cells, inhibition of ACC not only reduces de novo lipogenesis (DNL), but also suppresses proliferation and impairs mitochondrial fitness, indicating that ACC is both a rate-limiting node in fatty acid synthesis and an important metabolic inflection point [54].

FASN is located at the terminal step of de novo fatty acid synthesis and is responsible for generating palmitic acid, the most basic saturated fatty acid backbone [55]. In GBM, FASN-related lipogenic programs are closely associated with malignant progression, and the recently reported FGFR3-FASN interaction suggests that in some recurrent epithelioid GBMs, the FASN node can be abnormally activated through structural rearrangement [56]. Related studies have also shown that FASN is highly expressed in GBM and is associated with tumor grade; inhibition of FASN blocks lipid synthesis and induces cell-cycle arrest and apoptosis in glioma cells [57,58,59]. For example, targeting FASN with cerulenin reduces glioma cell proliferation and increases apoptosis markers [59]. Inhibition of FASN also downregulates stem cell markers such as SOX2, Nestin, and CD133 [49], suggesting that it is also crucial for maintaining glioma stemness. In addition, Zhang W et al. reported that FASN can enhance stemness and the inflammatory microenvironment by promoting EGFR palmitoylation and activating STAT1 signaling, thereby recruiting pro-oncogenic macrophages [60]. FASN plays multiple pro-tumor roles in GBM and is a potential therapeutic target.

If FASN determines whether the SFA backbone can be synthesized, then SCD1 and the brain-specific isoform SCD5 determine whether these SFAs can be promptly converted into MUFAs, a step that is particularly critical in GBM. SCD1 and SCD5 are responsible for converting saturated fatty acids into MUFAs. In GBM, SCD1/5 are generally upregulated, increasing MUFA content in membranes and improving membrane fluidity and signaling efficiency [20]. Recent studies suggest that SCD5 preferentially desaturates C18:0 and participates in lipid remodeling [61]. GBM does not rely solely on SCD1, but also uses SCD1/SCD5 together to maintain the MUFA pool and membrane lipid homeostasis. Inhibition of SCD1 leads to phospholipid remodeling and cytotoxicity, and can reduce TMZ resistance through the Akt/GSK3β/β-catenin axis [20]. Overall, SCD activity maintains endogenous MUFA supply, and SCD inhibitors have been shown to suppress glioma cell proliferation and resistance to combination therapy [62].

Parallel to the SFA-to-MUFA pathway is the PUFA synthesis branch mediated by FADS1/2 and ELOVL5/2. FADS1/2 introduce double bonds during PUFA synthesis, whereas ELOVL5/ELOVL2 extend the carbon chain to generate long-chain fatty acids. FADS and ELOVL determine long-chain PUFA levels, and cancer cell studies have shown that FADS2 can help cells survive through endoplasmic reticulum stress-related adaptive mechanisms when SCD1 is inhibited, while ELOVL5 can support proliferation and invasion by promoting PUFA elongation [63]. Therefore, they are likely involved in regulating the PUFA pool, membrane composition, and treatment response in GBM. Studies have found that inhibition of FADS2 or ELOVL2 blocks PUFA generation and leads to accumulation of the saturated fatty acid palmitic acid, thereby enhancing TMZ efficacy [31,64]. Chen Z et al. noted in other tumors that a shift from the conventional SCD1-dependent MUFA synthesis pathway to FADS2 products (such as n-10 MUFAs) is associated with increased tumor invasiveness [65].

This entire enzymatic system ultimately determines the intracellular ratio of SFA, MUFA, and PUFA in tumor cells, and changes in this ratio further affect membrane fluidity, endoplasmic reticulum homeostasis, DNA damage repair, and treatment sensitivity; at the phenotypic level, these changes can be summarized as alterations in proliferation, adhesion/invasion, stemness maintenance, and drug resistance (Table 1) [66].

## 4. Imbalanced Unsaturated/Saturated Fatty Acid Ratios Reshape Membrane Microdomains and Drive Signal Transduction and Cell Dynamics

**Figure 1:**
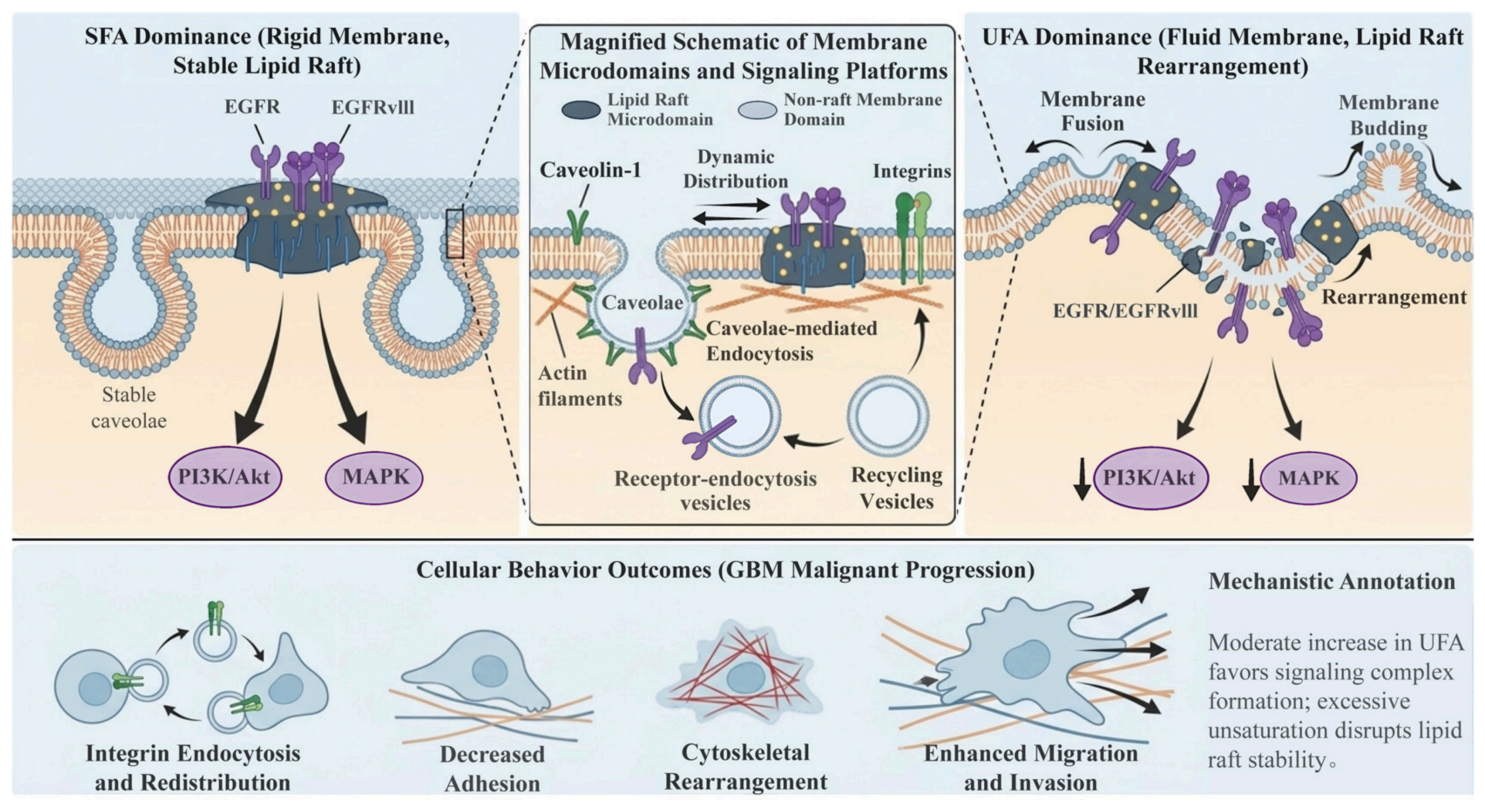
UFA/SFA ratio reshapes membrane microdomains and signal transduction. Schematic illustration showing how alterations in the unsaturated-to-saturated fatty acid (UFA/SFA) ratio modulate plasma membrane organization and downstream cellular behaviors in GBM. Changes in fatty acid composition influence lipid raft/microdomain stability, thereby regulating the spatial distribution and clustering of receptors such as EGFR/EGFRvIII and integrins. These membrane structural dynamics further modulate oncogenic signaling pathways (e.g., PI3K/Akt, MAPK), endocytosis, and cytoskeletal remodeling, ultimately affecting glioma cell proliferation, invasion, stemness maintenance, and therapeutic response. SFA, saturated fatty acid; UFA, unsaturated fatty acid.

The unsaturated/saturated fatty acid ratio (UFA/SFA) is not merely a metabolic readout, but an important determinant of the transition of cell membranes from a liquid-ordered to a liquid-disordered state, and it also serves as a key bridge linking metabolism to signal transduction [23,67]. This effect depends not only on the overall degree of unsaturation, but also on the number and position of double bonds, as well as the position of the first double bond in the carbon chain, all of which further alter membrane thickness, flexibility, permeability, and local curvature [68]. SFA more readily forms liquid-ordered lipid rafts together with cholesterol and sphingolipids, whereas UFA, because of the “kink” introduced by double bonds, weakens tight lipid packing, increases membrane deformability, and promotes membrane bending, fusion, and microdomain remodeling. Therefore, changes in the UFA/SFA ratio directly affect the stability of membrane microdomains and the spatial organization of receptors [23,69]. Within this framework (Figure 1), membrane microdomains constitute the structural basis through which the UFA/SFA ratio exerts its effects [70].

These cholesterol- and sphingolipid-rich microdomains can act as signaling platforms to enrich receptors and downstream effector molecules, thereby determining the spatial organization of signal transduction [71,72]. In glioblastoma, studies have shown that EGFR and its mutant EGFRvIII are closely associated with caveolin-1/caveolae on the cell membrane surface, and that caveolin-1 is upregulated in tumor tissue, suggesting that GBM cells may maintain aberrant receptor signaling by regulating membrane microdomains [73]. Alterations in membrane fatty acid saturation can influence receptor organization by modifying lipid packing, membrane order, and the stability of cholesterol-rich microdomains. These changes may affect the spatial clustering, activation threshold, and endocytosis dynamics of membrane receptors, thereby influencing downstream signaling intensity. In this context, changes in the UFA/SFA ratio directly affect lipid raft stability and receptor distribution on the membrane, thereby modulating the activation strength of key pathways such as PI3K/Akt and MAPK [4,71]. Notably, the influence of different degrees of unsaturation on signaling is nonlinear: a moderate increase in UFA facilitates lateral diffusion of membrane proteins and complex formation, whereas excessive unsaturation may disrupt lipid raft structure and cause disassembly of signaling platforms [23,72]. This “balanced state” suggests that glioma does not simply pursue a high unsaturation level, but rather finely regulates the UFA/SFA ratio to optimize signaling [4].

Beyond receptor signaling, the UFA/SFA ratio further affects cell behavior by altering membrane mechanical properties [23]. Increased UFA reduces membrane rigidity and increases bending capacity, thereby promoting membrane budding, vesicle formation, and membrane fusion [23]. These changes directly influence endocytosis and recycling pathways, and endocytosis is itself an important mechanism for regulating the persistence and termination of receptor signaling. For example, integrin endocytosis and redistribution depend on changes in membrane microdomains and membrane curvature, which determine the strength of adhesion between cells and the extracellular matrix and thereby affect migration capacity [74]. In tumor cells, accelerated integrin turnover is usually associated with increased invasiveness; therefore, the UFA/SFA ratio indirectly regulates migration and invasion by affecting membrane dynamics [74,75], and this mechanism is of even greater significance in GBM [76,77]. Studies have shown that stress such as radiotherapy can induce glioma cells to accumulate UFA and form lipid droplets, thereby buffering endoplasmic reticulum stress and maintaining cell survival, suggesting that an increased UFA/SFA ratio is an adaptive survival response to therapy, which may paradoxically confer resistance while also increasing vulnerability to subsequent oxidative insults [77]. Meanwhile, excessive unsaturation can increase membrane lipid susceptibility to oxidative stress, indicating that glioma must maintain a dynamic balance between “signaling optimization” and “oxidative risk” [8]. Therefore, therapies that alter the UFA/SFA ratio, such as PUFA supplementation or SCD inhibition, could not only impact metabolism but also directly disrupt the physical integrity of membrane signaling hubs, providing a dual mechanism of action. Cross-tumor studies further support this view; for example, in ovarian cancer, SCD-mediated desaturation protects cells from saturated fatty acid-induced ER stress and apoptosis, whereas SCD inhibition suppresses tumor growth [78]. In addition, omega-3 PUFAs can disturb lipid raft structure and alter the localization of immune signaling complexes, thereby affecting immune responses [79,80].

The UFA/SFA ratio can therefore be regarded as a core hub linking “fatty acid metabolism–membrane structure–signal transduction–cell behavior”. By regulating membrane microdomains, membrane mechanics, and signaling platform stability, it ultimately influences GBM proliferation, invasion, and therapeutic response [4,81].

## 5. Fatty Acid Metabolism and the Glioma Immune Microenvironment

**Figure 2:**
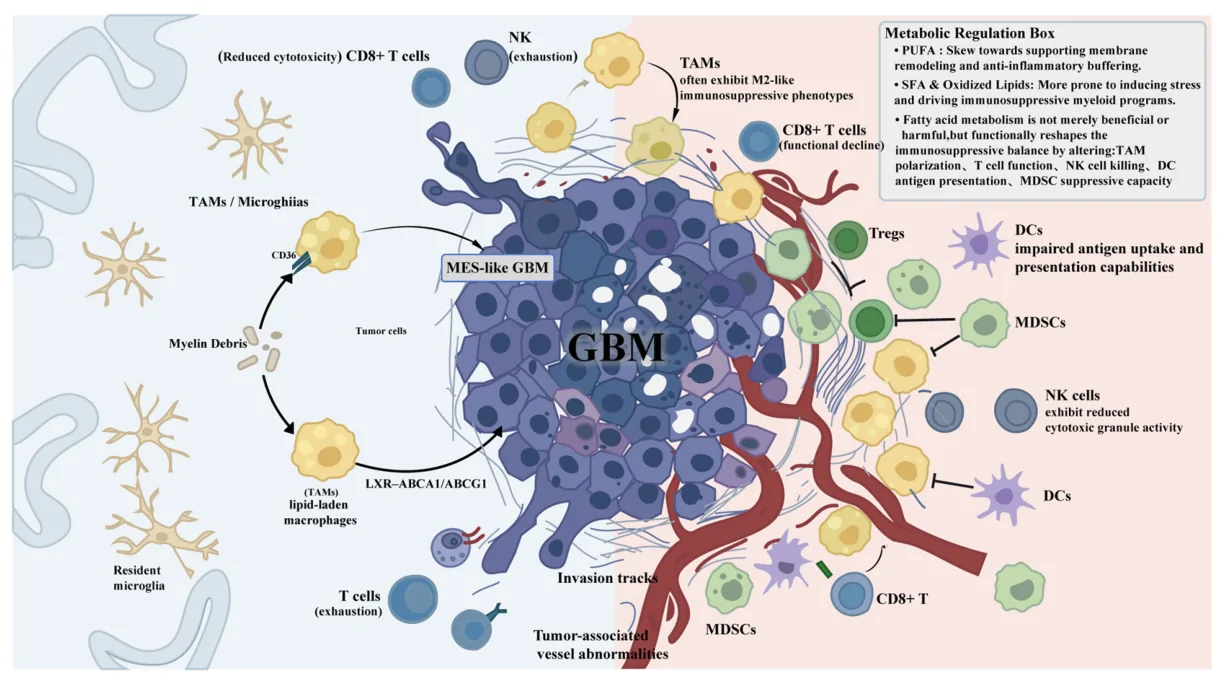
Fatty acid metabolism reshapes the GBM immune microenvironment. Schematic overview of metabolic crosstalk between glioblastoma cells and stromal/immune cells mediated by fatty acid metabolism. Tumor cells and tumor-associated macrophages (TAMs) engage in active lipid exchange (e.g., CD36-mediated uptake, LXR-ABCA1/ABCG1-dependent transfer), forming a “tumor-TAM-myelin” lipid cycle that reinforces an immunosuppressive phenotype. Differential fatty acid utilization— including fatty acid oxidation (FAO), lipid droplet formation, and altered fatty acid composition—differentially modulates the functional states of TAMs, CD8+ T cells, regulatory T cells (Tregs), natural killer (NK) cells, dendritic cells (DCs), and myeloid-derived suppressor cells (MDSCs), collectively tilting the immune balance toward suppression and tumor progression.

The immune microenvironment of GBM is composed of tumor cells, microglia, monocyte-derived tumor-associated macrophages (TAMs), a small number of lymphocytes, and antigen-presenting cells [82] (Figure 2). Recent single-cell and spatial multi-omics studies have further shown that GBM contains a TAM subpopulation enriched in lipid droplets, metabolically active, and more proliferative, especially in the mesenchymal subtype [83,84,85]. Recent studies have shown that TAMs can take up myelin debris via CD36 to form lipid-laden macrophages enriched in lipid droplets, and subsequently transfer myelin-derived lipids back to tumor cells via the LXR-ABCA1/ABCG1 axis, thereby enhancing MES-like GBM growth while maintaining an immunosuppressive phenotype. This “tumor-TAM-myelin” lipid cycle provides direct evidence for GBM immune–metabolic crosstalk [83,84,86]. From the perspective of tumor biology, fatty acid uptake, lipid droplet formation, and fatty acid oxidation (FAO) often drive TAMs toward an M2-like, immunosuppressive program and promote angiogenesis, tissue remodeling, and tumor invasion, while CD36, FABP4/5, and related lipid metabolic networks are key interfaces connecting exogenous lipids with macrophage functional reprogramming [86,87,88,89].

In addition to myeloid cells, fatty acid metabolism also affects CD8+ T cells. Under nutrient deprivation and hypoxic conditions, moderate fatty acid utilization and FAO can help effector T cells maintain function and, in some models, improve response to immunotherapy; however, lipid overload, abnormal fatty acid uptake, and persistent metabolic stress are more likely to drive T-cell dysfunction or exhaustion [90,91,92,93]. Regulatory T cells (Tregs) exhibit another pattern of lipid metabolic adaptation: they often rely more on FAO, fatty acid uptake, and pathways such as FABP5/CD36 to maintain survival and suppressive function within the tumor microenvironment, making lipid-rich environments more likely to favor immunosuppression and indirectly weaken anti-tumor immunity [90,94,95,96]. Natural killer (NK) cells are also strongly influenced by fatty acid metabolism. In the tumor microenvironment, lipid overload and oxidative stress impair their cytotoxic activity, whereas metabolic reprogramming may help NK cells maintain survival and killing capacity in harsh conditions. Thus, fatty acids are not only the “fuel” of tumor cells, but also a fundamental variable determining whether innate immune effector functions can operate normally [97,98]. Dendritic cells (DCs) and myeloid-derived suppressor cells (MDSCs) further amplify this effect: a high-fat state can disturb DC homeostasis and antigen-presenting capacity by enhancing mitochondrial FAO, while MDSCs often maintain immunosuppressive function by switching from glycolysis to FAO, thereby jointly weakening T-cell priming and the amplification of anti-tumor immunity [99,100,101].

PUFAs are generally more involved in membrane remodeling and inflammatory buffering, whereas excessive SFA more readily triggers metabolic stress responses and drives myeloid cells toward an immunosuppressive phenotype. However, this direction is clearly dependent on cell type and disease stage. Therefore, in GBM, a more appropriate interpretation is not that a certain class of fatty acid is absolutely beneficial or harmful, but rather that they collectively shape the balance between immunosuppressive and immune-activating states by altering TAM polarization, antigen presentation, effector T-cell function, and innate immune cell states [102,103,104]. Thus, the significance of fatty acids should not be limited to energy supply or structural lipids; they should be regarded as a common language regulating TAM education, antigen presentation, T-cell effector function, NK cell killing, and MDSC suppression. From a translational perspective, blocking fatty acid uptake, lipid droplet formation, or FAO, or reshaping the fatty acid composition to reduce immunosuppressive lipid signaling, may act synergistically with checkpoint inhibitors, myeloid-targeted therapies, and metabolic interventions.

## 6. Nutritional and Targeted Therapies for Fatty Acid Metabolism

Among nutritional interventions, ketogenic diet (KD) or ketogenic metabolic therapy (KMT) is the most established approach, whose core strategy is to reduce glucose availability and alter substrate supply in an attempt to amplify the metabolic vulnerability of tumor cells [105]. The KEATING randomized feasibility study, together with subsequent systematic reviews, clinical framework proposals, case reports, and clinical studies, suggests that these strategies are feasible and relatively safe in GBM, but the current clinical evidence remains exploratory overall, mainly reflecting feasibility, adherence, and biological signals rather than definitive survival benefit [106]. KMT remains worthy of further investigation because GBM does not rely solely on glucose for energy; experimental studies have shown that GBM can use fatty acids and ketone bodies to sustain growth under ketogenic conditions, and positron emission/isotopic tracing, metabolic flux analysis, and ketone-ester supplementation models further demonstrate that tumors rapidly switch substrate utilization according to the nutritional environment. Therefore, dietary intervention is more suitable as a “metabolic stress amplifier” and a sensitizer to standard therapy, rather than as a stand-alone replacement for surgery, radiotherapy, and TMZ [107,108]. This also explains why the current translational focus has shifted from “whether to use ketogenic therapy” to “how to use it precisely,” including patient selection, setting the intensity of fatty acid/ketone supply, monitoring the glucose-ketone index (GKI), and evaluating long-term tolerability, while integrating it into routine treatment workflows as much as possible [109]. In GBM, KMT is more like an adjuvant therapy that needs to match the individual metabolic background, rather than a one-size-fits-all approach.

Unlike the strategy of “restricting carbon sources,” a PUFA-rich modified diet is an approach that exploits metabolic vulnerability [28]. GBM is highly dependent on lipid uptake and storage, and exogenous PUFAs can “hijack” this metabolic pathway. After being incorporated into lipid droplets, PUFAs induce their breakdown, release free fatty acids, and trigger lipid peroxidation, thereby synergizing with radiotherapy to amplify cytotoxicity. This finding suggests that the key to nutritional therapy for brain tumors is not total fat content, but rather the type and proportion of fatty acids and their coupling with radiotherapy [8].

Among targeted pharmacologic strategies, ACC1/ACC2 and FASN represent key nodes in de novo fatty acid synthesis, because they determine the flux of acetyl-CoA into palmitic acid and subsequent membrane lipids; EGFRvIII GBM is particularly sensitive to ACC inhibition, and the FASN inhibitor TVB-2640/denifanstat has already entered a phase II clinical study in recurrent high-grade astrocytoma [110], indicating that “blocking fatty acid synthesis” is no longer merely conceptual but already has clinical translational feasibility. From the perspective of balancing fatty acid desaturation, SCD1 determines the efficiency of SFA-to-MUFA conversion, thus affecting membrane fluidity, endoplasmic reticulum homeostasis, lipotoxicity threshold, and response to TMZ [20,111]. In GBM, SCD1 inhibition leads to saturated fatty acid accumulation, increased ER stress, apoptosis, and impaired DNA repair, making this one of the most druggable desaturation nodes [20]. On the fatty acid catabolism side, GBM has a clear dependence on FAO. Carnitine palmitoyltransferase 1A (CPT1A) is the rate-limiting enzyme that governs entry of fatty acids into mitochondrial beta-oxidation and is a key gatekeeper of this pathway. The CPT1 inhibitor etomoxir combined with TMZ can reduce stemness and invasiveness of patient-derived tumorspheres (PDTs) and prolong survival in animals, thus FAO inhibition is considered a feasible strategy to overcome GBM metabolic adaptation and therapy resistance [112]. However, the pharmacological limitations of FAO inhibitors are also important: etomoxir has been shown in other cancer types to have obvious off-target and dose-limiting effects, so it is more appropriate to advance cautiously under biomarker and metabolic-phenotype guidance [113,114]. In contrast, the CPT1/CPT2 inhibitor perhexiline has shown the ability to cross the blood–brain barrier and produce FYN-dependent anti-tumor effects in GBM, suggesting that fatty acid-related drugs need not revolve only around CPT1 and can also be extended to the broader lipid metabolic network through drug repurposing [115]. Fatty acid uptake is also worth attention, because CD36 is not only a gatekeeper for exogenous long-chain fatty acids entering tumor and immune cells, but is also associated with metastasis initiation, lipid droplet accumulation, and immunosuppression; the GBM CD36/TSP-1/CD47 axis has been proposed as a translational node, and the CD36 inhibitor SMS121 has been shown in other myeloid tumors to block fatty acid uptake and reduce cell activity, providing a useful reference for blocking exogenous lipid entry in GBM [86].

Therefore, future strategies should move beyond single-agent metabolic intervention toward rational combinations of diet, targeted inhibitors, and standard-of-care therapy. Dietary remodeling, FASN/ACC/SCD/CPT1/CD36 inhibition, TMZ, radiotherapy, and anti-angiogenic therapy may be integrated according to tumor metabolic phenotype. Lipidomics-based assessment of the tumor UFA/SFA ratio, together with FASN and SCD1 expression levels, could serve as predictive biomarkers to stratify patients for metabolic therapy and to monitor adaptive resistance. However, clinical translation of metabolic targeting in GBM remains challenging due to limited drug delivery across the blood–brain barrier, substantial intratumoral heterogeneity, and the lack of validated biomarkers for patient stratification. Future studies integrating lipidomic profiling with molecular classification will be essential to identify patients most likely to benefit from metabolic interventions. Nanoparticles co-delivering PUFAs and SCD inhibitors may help overcome the blood–brain barrier and synergistically disrupt the UFA/SFA balance in situ, thereby improving intracranial exposure while concentrating metabolic pressure within the tumor microenvironment [116].

## 7. Conclusions and Future Directions

Fatty acid metabolic reprogramming in GBM has profound effects on its biological behavior and therapeutic response. The dynamic balance among MUFAs, PUFAs, and SFAs not only affects membrane lipid composition, signal transduction, and mechanical properties of glioma cells, but also participates in their proliferation, migration/invasion, stemness maintenance, cell death, therapy resistance, and remodeling of the immune microenvironment. In particular, the UFA/SFA ratio has emerged as an integrative indicator of lipid metabolic reprogramming and provides a useful framework for understanding malignant progression in GBM. Although the UFA/SFA ratio conceptually reflects the balance between lipid saturation and desaturation, a standardized quantitative UFA/SFA index has not yet been established in human GBM. Current lipidomic studies primarily quantify individual fatty acids or lipid subclasses rather than a unified UFA/SFA ratio, and differences in tissue sampling, lipid classes analyzed, and analytical methodologies have hindered direct quantitative comparison across studies. Nevertheless, current evidence generally supports an overall shift toward increased lipid unsaturation in GBM, suggesting that the biological significance of the UFA/SFA ratio currently lies in reflecting the dynamic state of lipid remodeling rather than a fixed numerical threshold. Future standardized and spatially resolved lipidomic analyses may facilitate the establishment of clinically comparable UFA/SFA metrics.

Beyond its biological significance, the UFA/SFA ratio also shows potential as a metabolic biomarker for GBM. Tissue-based lipidomic profiling is technically feasible, whereas its application in liquid biopsy remains exploratory because evidence from blood or cerebrospinal fluid is still limited. However, several barriers currently prevent clinical implementation, including marked intratumoral heterogeneity, the lack of standardized lipidomic workflows and diagnostic cut-off values, and the absence of large prospective clinical validation studies. Therefore, further methodological standardization and multicenter validation will be essential before the UFA/SFA ratio can be translated into routine clinical testing.

Future studies should further move from “descriptive correlations” to “causal mechanism dissection” and “clinical translation validation.” On the one hand, spatial lipidomics, single-cell multi-omics, and metabolic flux analysis are needed to define fatty acid profile differences across different regions, molecular subtypes, and cell populations in glioma, especially the spatial heterogeneity of the UFA/SFA ratio at the tumor margin, necrotic core, stem-like cell populations, and immune cell populations. On the other hand, more refined functional validation systems are needed around key enzymes and key fatty acids to clarify how fatty acid metabolism determines tumor fate through pathways such as membrane microdomains, oxidative stress, ferroptosis, and immune regulation. More importantly, interventions targeting fatty acid metabolism should be combined as much as possible with current standard therapy, immunotherapy, and radiotherapy/chemotherapy, and patients who truly benefit should be selected based on metabolic stratification, thereby promoting this field from basic research to precision medicine.

The significance of fatty acid metabolism in GBM is no longer limited to “energy supply” or “membrane construction”; rather, it has risen to a comprehensive regulatory network connecting metabolism, membrane biology, cell fate, and the immune ecosystem. Research built around this network will provide new theoretical foundations and intervention strategies for GBM stratification and combined diagnosis and treatment.

## Figures and Tables

**Table 1:** Key fatty acid metabolic enzymes and their biological roles in GBM.

Metabolic Module	Key Enzymes	Major Function	Role in GBM	Representative Inhibitors
Acetyl-CoA supply and de novo lipogenesis	ACLY, ACC1/2, FASN	Convert citrate to acetyl-CoA and promote de novo fatty acid synthesis	Support tumor growth, proliferation, membrane biosynthesis and stemness maintenance	BMS-303141 (ACLY) ND-646 (ACC) TVB-2640 (FASN)
Fatty acid desaturation	SCD1, SCD5	Convert saturated fatty acids into monounsaturated fatty acids (MUFAs) and maintain membrane lipid homeostasis	Increase membrane fluidity, maintain ER homeostasis, promote stemness and therapy resistance	CAY10566, A939572
PUFA synthesis and elongation	FADS1/2, ELOVL2/5	Catalyze PUFA desaturation and elongation	Regulate PUFA pools, membrane composition, adaptive metabolism and TMZ response	SC-26196 (FADS2)
Fatty acid β-oxidation	CPT1A	Rate-limiting enzyme controlling mitochondrial fatty acid oxidation	Supports metabolic adaptation, invasion, stemness and therapy resistance	Etomoxir, Perhexiline
Ferroptosis-associated lipid remodeling	ACSL4	Incorporates PUFAs into membrane phospholipids and regulates ferroptosis sensitivity	Determines susceptibility to lipid peroxidation and ferroptotic cell death	Triacsin C *

* Triacsin C is a broad-spectrum ACSL inhibitor and is not specific for ACSL4.

## Data Availability

No new data were created or analyzed in this study. Data sharing is not applicable to this article.

## Abbreviations

The following abbreviations are used in this manuscript:

AA	Arachidonic Acid
ABCA1	ATP-Binding Cassette Transporter A1
ABCG1	ATP-Binding Cassette Transporter G1
ACC	Acetyl-CoA Carboxylase
ACLY	ATP Citrate Lyase
ACSL4	Acyl-CoA Synthetase Long-Chain Family Member 4
Akt	Protein Kinase B
CD36	Cluster of Differentiation 36
COX-2	Cyclooxygenase-2
CPT1A	Carnitine Palmitoyltransferase 1A
DCs	Dendritic Cells
DHA	Docosahexaenoic Acid
EGFR	Epidermal Growth Factor Receptor
EGFRvIII	Epidermal Growth Factor Receptor Variant III
ELOVL	Elongation of Very Long Chain Fatty Acids
FABP7	Fatty Acid-Binding Protein 7
FAO	Fatty Acid Oxidation
FADS1/2	Fatty Acid Desaturase 1 and 2
FASN	Fatty Acid Synthase
GBM	Glioblastoma
GKI	Glucose-Ketone Index
GPX4	Glutathione Peroxidase 4
KD	Ketogenic Diet
KMT	Ketogenic Metabolic Therapy
LXR	Liver X Receptor
MAPK	Mitogen-Activated Protein Kinase
MDSCs	Myeloid-Derived Suppressor Cells
MUFA	Monounsaturated Fatty Acid
mTORC1	Mechanistic Target of Rapamycin Complex 1
NK	Natural Killer
PA	Palmitic Acid
PI3K	Phosphoinositide 3-Kinase
PPARγ	Peroxisome Proliferator-Activated Receptor gamma
PUFA	Polyunsaturated Fatty Acid
ROS	Reactive Oxygen Species
SCD1	Stearoyl-CoA Desaturase 1
SCD5	Stearoyl-CoA Desaturase 5
SFA	Saturated Fatty Acid
SOAT1	Sterol O-Acyltransferase 1
SOX2	SRY-Box Transcription Factor 2
SREBP-1	Sterol Regulatory Element-Binding Protein 1
STAT1	Signal Transducer and Activator of Transcription 1
TAMs	Tumor-Associated Macrophages
TMZ	Temozolomide
Tregs	Regulatory T Cells
UFA	Unsaturated Fatty Acid
